# Erlotinib-Conjugated Iron Oxide Nanoparticles as a Smart Cancer-Targeted Theranostic Probe for MRI

**DOI:** 10.1038/srep36650

**Published:** 2016-11-11

**Authors:** Ahmed Atef Ahmed Ali, Fei-Ting Hsu, Chia-Ling Hsieh, Chia-Yang Shiau, Chiao-Hsi Chiang, Zung-Hang Wei, Cheng-Yu Chen, Hsu-Shan Huang

**Affiliations:** 1 Molecular and Cell Biology, Taiwan International Graduate Program, Institute of Molecular Biology, Academia Sinica, Taipei 115, Taiwan; 2 Graduate Institute of Life Sciences, National Defense Medical Center, Taipei 114, Taiwan; 3 Department of Medical Imaging, Taipei Medical University Hospital, Taipei 110, Taiwan; 4 Translational Imaging Research Center, College of Medicine, Taipei Medical University, Taipei 110, Taiwan; 5 The Ph.D. Program for Translational Medicine, College of Medical Science and Technology, Taipei Medical University, Taipei 110, Taiwan; 6 Graduate Institute of Medical Sciences, National Defense Medical Center, Taipei 114, Taiwan; 7 Department of Power Mechanical Engineering, National Tsing Hua University, Hsinchu 300, Taiwan; 8 Graduate Institute for Cancer Biology & Drug Discovery, College of Medical Science and Technology, Taipei Medical University, Taipei 110, Taiwan

## Abstract

We designed and synthesized novel theranostic nanoparticles that showed the considerable potential for clinical use in targeted therapy, and non-invasive real-time monitoring of tumors by MRI. Our nanoparticles were ultra-small with superparamagnetic iron oxide cores, conjugated to erlotinib (FeDC-E NPs). Such smart targeted nanoparticles have the preference to release the drug intracellularly rather than into the bloodstream, and specifically recognize and kill cancer cells that overexpress EGFR while being non-toxic to EGFR-negative cells. MRI, transmission electron microscopy and Prussian blue staining results indicated that cellular uptake and intracellular accumulation of FeDC-E NPs in the EGFR overexpressing cells was significantly higher than those of the non-erlotinib-conjugated nanoparticles. FeDC-E NPs inhibited the EGFR–ERK–NF-κB signaling pathways, and subsequently suppressed the migration and invasion capabilities of the highly invasive and migrative CL1-5-F4 cancer cells. *In vivo* tumor xenograft experiments using BALB/c nude mice showed that FeDC-E NPs could effectively inhibit the growth of tumors. T_2_-weighted MRI images of the mice showed significant decrease in the normalized signal within the tumor post-treatment with FeDC-E NPs compared to the non-targeted control iron oxide nanoparticles. This is the first study to use erlotinib as a small-molecule targeting agent for nanoparticles.

Epidermal growth factor receptor (EGFR) is a transmembrane glycoprotein having tyrosine kinase activity that affects several critical signaling pathways related to cancer cell growth, apoptosis, angiogenesis, aggressiveness and invasiveness. EGFR is overexpressed in a large number of solid tumors including lung, colorectal, breast, ovarian, and head and neck cancers. Such increased activity of the receptor is correlated with poor response to therapy^1^,^2^,^3^,^4^. One of the most effective targeting strategies to inhibit EGFR is the use of small-molecule tyrosine kinase inhibitors such as erlotinib, which have proved to be highly selective for the EGFR tyrosine kinase, resulting in cell cycle arrest, inhibition of proliferation and apoptosis of cancer cells^4^,^5^,^6^,^7^. The significant variation in response to erlotinib treatment among patients^8^ as well as the acquired resistance that emerges during the course of treatment^9^ require diagnostic tools to classify and identify tumor types that will benefit from the treatment, and to monitor the treatment response regularly during the treatment period. One important non-invasive technique used in clinical practice for diagnosis, grading, staging and follow-up of cancer is magnetic resonance imaging (MRI). MRI requires the use of contrast probes with desired properties such as iron oxide, manganese oxide, gold, silver and gadolinium nanoparticles^10^.

Advances in diagnostic imaging capabilities as well as in targeted drug delivery have resulted in the development of new “theranostic” nanoparticle platforms with therapeutic and diagnostic properties. Among the molecules used to impart targeting capabilities to the drug-carrying nanoparticles are monoclonal antibodies, peptides, aptamers, and small-molecules. Small-molecules exhibit great promise in the field of targeted anticancer nanoparticle therapeutics compared to other classes of targeting molecules due to their small size, diverse structures, stability and low cost of production, which makes them more suitable and feasible for clinical applications^11^,^12^. While designing our nanoparticle formulation presented in this study, we aimed to use erlotinib for its dual properties as a therapeutic drug and targeting agent because of the advantages exhibited by the small-molecules over other targeting agents. Also, we aimed to use iron oxide as the MRI contrast agent because it is superparamagnetic, biocompatible, biodegradable and inexpensive^13^,^14^.

Here, we present a smart targeted therapeutic formulation of ultra-small superparamagnetic iron oxide nanoparticles conjugated to erlotinib (FeDC-E NPs) as a novel theranostic biomarker that can be monitored by MRI. Interestingly, FeDC-E NPs showed a smart preferential release of the drug intracellularly rather than into the blood or body fluids as tested by mimic fluids. Potent therapeutic efficacy and significant targeting capability of FeDC-E NPs were confirmed by cell viability experiments, TEM imaging, Prussian blue staining, and MRI. In addition, FeDC-E NPs significantly suppressed the invasion and migration capabilities of the highly invasive and migrative CL1-5-F4 cancer cells more than erlotinib. Moreover, FeDC-E NPs inhibited phosphorylation of the EGFR as well as the EGFR–ERK–NF-κB signaling pathways of the EGFR overexpressing cells along with the expression of the downstream tumor promoting proteins MMP-9 and XIAP. *In vivo* experiments of BALB/c nude mice bearing xenografts of CL1-5-F4 cells revealed that FeDC-E NPs significantly inhibited tumor growth compared to the control groups. T_2_-weighted MRI images of the mice showed significant drop in the normalized signal within the tumor post-treatment with FeDC-E NPs compared to the non-targeted iron oxide control nanoparticles. In short, results of this study present a foundation for further translation of the FeDC-E NPs into clinical applications such as diagnosing cancer, and predicting and monitoring the treatment response of cancer patients.

## Results and Discussion

### Structural, physical and chemical characterization of the synthesized nanoparticles

**Figure 1:**
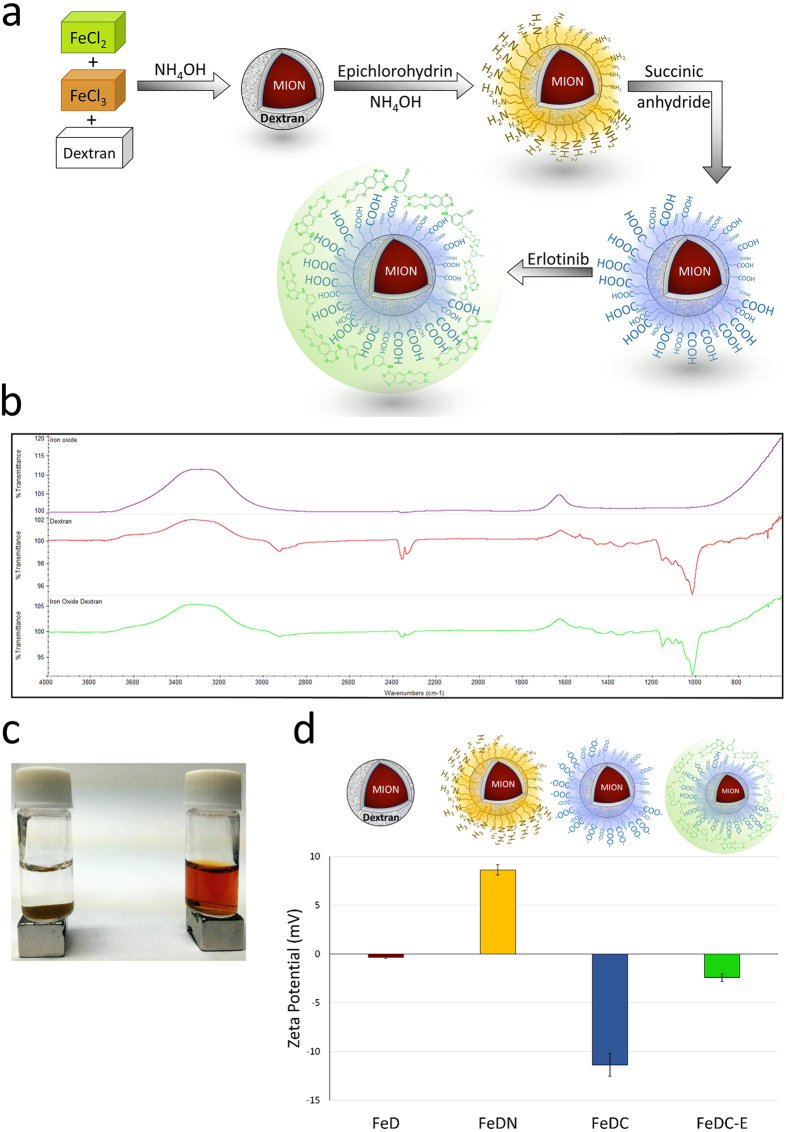
Synthesis and characterization of the nanoparticles (NPs). **(a)** Synthesis stages of the erlotinib-conjugated dextran-coated monocrystalline iron oxide nanoparticles (FeDC-E NPs). **(b)** FT-IR spectra of the non-dextran-coated iron oxide nanoparticles (Iron oxide; violet), dextran (Dextran; red) and the dextran-coated monocrystalline iron oxide nanoparticles (FeD NPs) (Iron Oxide Dextran; green). **(c)** Image of the FeD NPs (right) and the non-dextran-coated iron oxide nanoparticles (left) one week after placing over strong rare earth magnets. Physical stability of the FeD NPs was evident from absence of precipitation and aggregation towards the magnet due to stabilization by dextran coating. **(d)** Change in the surface charges of nanoparticles during the stages of synthesis. FeD: dextran-coated monocrystalline iron oxide nanoparticles (MION); FeDN: amino group-functionalized dextran-coated MION; FeDC: carboxyl group-functionalized dextran-coated MION; FeDC-E: erlotinib-loaded dextran-coated MION.

One of the main challenges that faced us while synthesizing the nanoparticles was retaining the biological activity of erlotinib after conjugation to the nanoparticles. We synthesized monocrystalline iron oxide nanoparticles (MION) using the alkaline co-precipitation method^15^,^16^,^17^ followed by crosslinking of thin dextran coating on the surface of nanoparticles. The surface of nanoparticles was then functionalized with amino groups that were modified later to carboxyl groups and conjugated to erlotinib. Details of the synthesis process are presented in the methods section and illustrated in Fig. 1a. In order to confirm the coordination of dextran with the synthesized MION, we obtained the FT-IR spectra of the non-dextran-coated nanoparticles, dextran and dextran-coated iron oxide nanoparticles (FeD NPs) (presented in Fig. 1b). Spectrum of the non-dextran-coated nanoparticles showed absorption peaks around 3300 and 1600 cm^−1^ indicating the O-H stretching and H-O-H bending of the physically adsorbed water on the particle surface as well as a broad absorption band at 600 cm^−1^ due to the Fe-O vibrations. The spectrum of the dextran showed the water O-H stretching and H-O-H bending peaks around 3300 and 1600 cm^−1^ as well as C-H stretching peak around 2900 cm^−1^ and strong C-O peak at 1000 cm^−1^ characteristic of the α-glucopyranose ring of dextran. The spectrum of the FeD NPs showed the characteristic peaks of dextran, especially in the fingerprint region with slight changes in the intensities and features of peaks, this is due to the coordination of dextran with the iron oxide on the nanoparticles surface. The FT-IR spectra we obtained for the nanoparticles are in concordance with previous reports^15^,^16^.

Physical stability and aggregation potential of nanoparticles were tested by placing the nanoparticle solutions over a strong rare earth magnet for one week and examining the solutions visually for any precipitate or aggregates. As shown in Fig. 1c, the non-dextran-coated iron oxide nanoparticles showed precipitation and aggregation of all particles, while the FeD NPs did not show any precipitation or aggregation and remained fully dispersed as a clear reddish brown solution, indicating strong physical stability due to dextran coating. However, applying an external magnetic field could move the whole nanoparticle solution in the tube (see Supplementary Video S1). To further test the effectiveness of dextran stabilization to our final product, we stored the FeDC-E NPs solution in tightly sealed glass tube for one year at 25 °C. Visual and microscopical examination of the solution at the end of the storage period showed that the nanoparticles were still fully dispersed with no aggregation or precipitation of the particles. This physical stability due to dextran coating is further illustrated in the transmission electron microscope (TEM) imaging results.

Surface charges of the nanoparticles determined by the zeta potential measurements are presented in Fig. 1d. FeD NPs showed a surface charge close to zero of −0.3 ± 0.1 mV. Amino group functionalization of the nanoparticles (amino group-functionalized dextran-coated iron oxide nanoparticles (FeDN NPs)) reversed the zeta potential of the FeD NPs to a positive value of 8.6 ± 0.5 mV due to the formation of positive (-N^+^H_3_) groups on the surface of nanoparticles in aqueous solutions, while the carboxyl group functionalization of nanoparticles (carboxyl group-functionalized dextran-coated iron oxide nanoparticles (FeDC NPs)) reversed the zeta potential of the FeDN NPs to a high negative value of −11.4 ± 1.2 mV due to the formation of negative (-COO^−^) groups on the surface of FeDC NPs in aqueous solutions. Conjugating the erlotinib base to the nanoparticles (FeDC-E NPs) reduced the negative charge of the FeDC NPs to a value of −2.4 ± 0.4 mV due to neutralization of the FeDC NPs carboxyl groups by the N atoms of the erlotinib base that may react in an acid-base fashion producing stable erlotinib-loaded nanoparticles. We designed this method of conjugating erlotinib to the nanoparticles, inspired by the HCl salt of erlotinib that is used in clinical practice, in order to retain the biological activity of erlotinib in the FeDC-E NPs formulation, because other methods of covalent linking will change the chemical structure of the drug and encumber its biological activity^18^.

Microwave plasma - atomic emission spectrometry (MP-AES) analysis showed that the iron content of the FeDC-E NPs solution is 1.913 mg/mL. The amount of erlotinib loaded in the FeDC-E NPs aqueous solution determined by the high-performance liquid chromatography (HPLC) is 151.32 μg/mL (Supplementary Fig. S2), while the aqueous solubility of the erlotinib base which we used in the synthesis of FeDC-E NPs is 14.02 μg/mL^19^. It is obvious from these results that our strategy to conjugate erlotinib to the MION was very effective, as it could load large amounts of erlotinib in the nanoparticles (more than ten-fold of its aqueous solubility).

### Morphology of the synthesized nanoparticles

**Figure 2:**
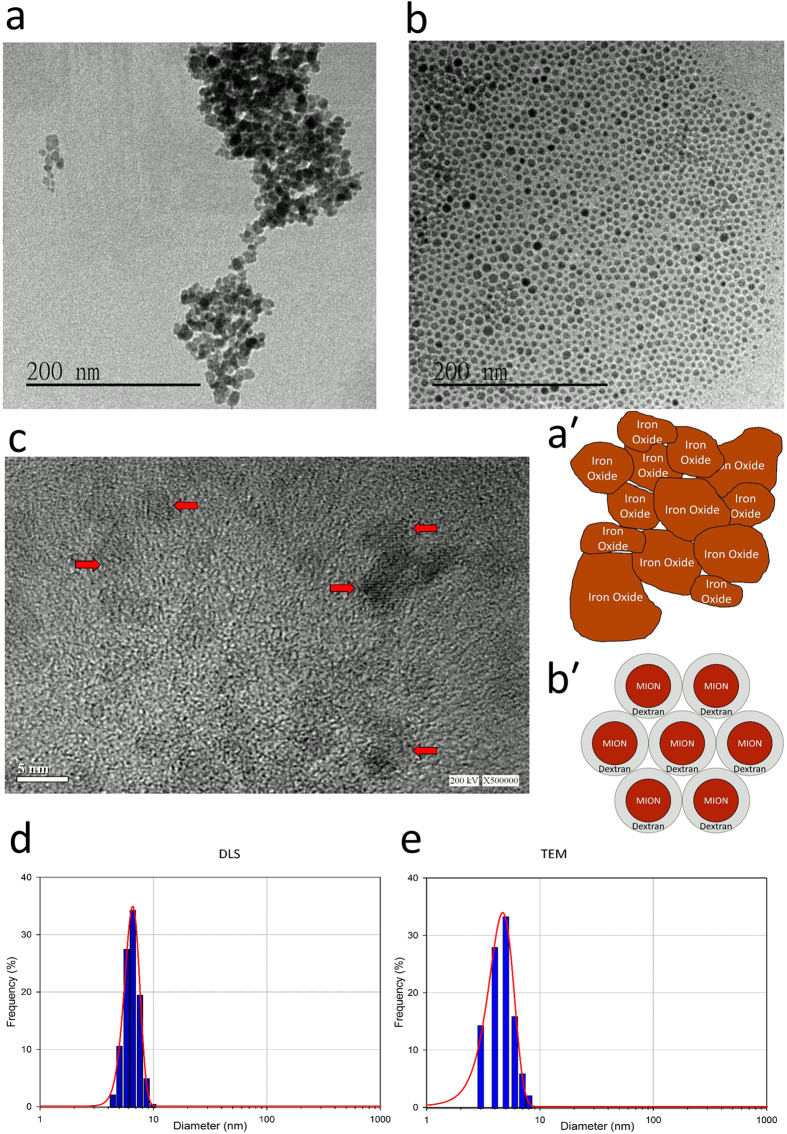
Morphology of nanoparticles. Transmission electron microscopy (TEM) images of **(a)** non-dextran-coated iron oxide nanoparticles and **(b)** dextran-coated monocrystalline iron oxide nanoparticles (MION), with their diagrammatic representations (**(a′)** non-dextran-coated iron oxide nanoparticles and **(b′)** dextran-coated MION) illustrating the aggregation/non-aggregation and uniformity/non-uniformity of shape of nanoparticles. **(c)** High resolution TEM (HRTEM) image of the dextran-coated MION showing the crystal lattice planes of the individual particles confirming the monocrystalline property of the nanoparticles, red arrows point to the lattice planes. **(d,e)** Size distribution of the MION obtained from the dynamic light scattering (DLS) **(d)** and TEM images **(e)**. DLS showed the diameter of nanoparticles to be 6.06 ± 0.9 nm, while TEM images showed nanoparticles of 4.28 ± 1.1 nm diameter. Red line curves denote log-normal fit with narrow size distribution.

TEM images showed that the non-dextran-coated iron oxide nanoparticles were aggregated with irregular shapes and sizes (Fig. 2a). Conversely, coating the MION with dextran produced fully dispersed, non-aggregated nanoparticles of regular spherical shape and smaller sizes than the non-dextran-coated nanoparticles as evident from Fig. 2b. High resolution TEM (HRTEM) imaging of the dextran-coated MION showed clear regular fringes of the crystal lattice planes of the individual particles indicating that the nanoparticles are monocrystalline (Fig. 2c). The FeDC-E NPs showed monodisperse isotopic-shaped particles with an iron oxide core of 4.28 ± 1.1 nm diameter and polydispersity index (PDI) of 0.07 as determined by the TEM images and calculated using ImageJ image analysis software. The hydrodynamic sizes of the FeDC-E NPs determined by the dynamic light scattering (DLS) were in agreement with those obtained from the TEM images, where the nanoparticles showed narrow log-normal size distribution curve of 6.06 ± 0.9 nm diameter and PDI of 0.02 (Fig. 2d,e). These results denote the presence of a thin dextran coating layer of about 1–2 nm around the MION core, such small-sized nanoparticles are desirable and have been reported once by Kumar *et al*. who used the same principle of nanoparticle synthesis (alkaline co-precipitation method)^16^. We achieved such properties by setting up favorable synthesis conditions, such as using high concentrations of dextran in the synthesis process as well as performing the synthesis reaction on ice to slow down the reaction rate. These conditions led to the adsorption of dextran instantaneously on the surface of the newly formed MION confining the space around the nanoparticles for further growth or aggregation of the nanoparticles, resulting in the production of stable ultra-small-sized MION that are fully dispersed in the solution. Furthermore, the dextran coating on the surface of the nanoparticles provides steric hindrance that prevents the MION from aggregation during further synthesis stages and shelf life (refer to Fig. 2a′ for illustration).

### Targeting and therapeutic capabilities of the nanoparticles

Theranostic probes are characterized by having targeting, therapeutic and imaging capabilities^12^. To investigate the targeting capability, and therapeutic efficiency of the FeDC-E NPs compared to the erlotinib drug, we tested the viability of two types of cells using the WST-1 assay following designated treatments. We used CL1-5-F4 cells that overexpress the EGFR^20^ and Jurkat cells that do not express the EGFR^21^ for these experiments. The CL1-5-F4 cells were generated from the CL1 human lung adenocarcinoma cells by five repeated *in vitro* selection processes of the invasive sublines generating the CL1-5 subline which was 6-fold more invasive than the parent cells^22^, then by four repeated *in vivo* selections of the highly invasive and migrative cells from the lungs of severe combined immunodeficient (SCID) mice generating the CL1-5-F4 subline^23^. Jurkat T-cell lymphoma cells are frequently used as EGFR-negative control cells in studies of various types of cancer^21^,^24^,^25^.

**Figure 3:**
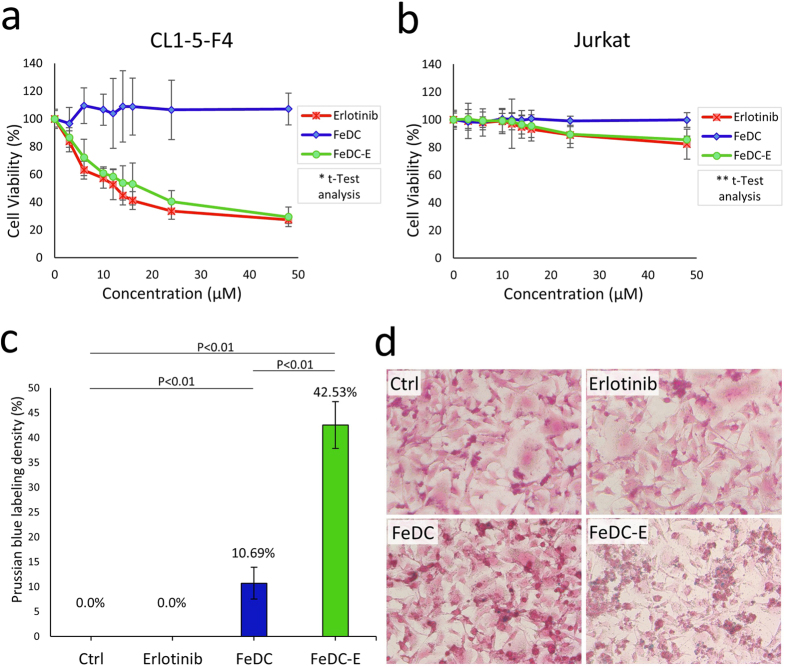
Therapeutic and targeting capabilities of the nanoparticles. Dose response curves of the cytotoxic activities of Erlotinib, carboxyl group-functionalized dextran-coated iron oxide nanoparticles (FeDC NPs) and erlotinib-loaded dextran-coated iron oxide nanoparticles (FeDC-E NPs) against the EGFR overexpressing CL1-5-F4 cells **(a)** and the EGFR negative Jurkat cells **(b)**. FeDC-E NPs retained the activity of erlotinib and exhibited selective cytotoxic effects against the EGFR overexpressing cancer cells, but not against the other cells. N.B. FeDC NPs formulation was tested using the same volume and iron concentration as the FeDC-E NPs used in the test (because FeDC NPs does not contain erlotinib); however, the scale and label of the X-axis of the FeDC NPs-treated cells were retained for the sake of comparison among tested groups. **(c**,**d)** Targeting capability of the nanoparticles detected using the Prussian blue experiments. **(c)** Percentages of the iron-labeled CL1-5-F4 cells quantified using the ImageJ software. Results showed a 4-fold increase in the cellular uptake of the targeted FeDC-E NPs compared to the non-targeted FeDC NPs due to the targeting capability of erlotinib to EGFR imparted to the FeDC-E nanoparticles. The P-values were calculated using the t-Test method assuming unequal variances. **(d)** Micrographs of CL1-5-F4 cells processed with the Prussian blue staining procedures. Uptaken nanoparticles are observed as blue spots. *, **Statistical t-test analysis showed non-significant difference among the cytotoxic activities of erlotinib and FeDC-E NPs for any of the tested concentrations for either of the tested cell lines (similar cytotoxic profiles for both treatments), whereas the cytotoxic activities of erlotinib and FeDC-E NPs against CL1-5-F4 cells showed statistical significant difference from their activities against Jurkat cells, and the cytotoxic activities of FeDC NPs did not show statistical significant difference among CL1-5-F4 and Jurkat cells.

To validate our choice of cell lines to be tested regarding the EGFR status, we tested the sensitivity of both cell lines to erlotinib. Results showed that erlotinib exerted a cytotoxic effect on CL1-5-F4 cells in a dose dependent manner. However, erlotinib did not suppress the viability of Jurkat cells even at very high doses. This is due to the overexpression of EGFR on CL1-5-F4 cells and its absence on Jurkat cells. Next, we tested the cytotoxicity of FeDC NPs (carboxyl group-functionalized dextran-coated nanoparticles without erlotinib) on both cell lines. Results showed that FeDC NPs did not exhibit any cytotoxicity against either cell line, highlighting the biocompatible nature of the nanoparticles. Then, we tested the cytotoxicity of the erlotinib-conjugated FeDC-E NPs against both cell lines. FeDC-E NPs exhibited cytotoxicity pattern similar to erlotinib, where the FeDC-E NPs inhibited viability of CL1-5-F4 cells but not Jurkat cells, effects on both cell lines were statistically significantly different. Collective results of the cell viability assays are presented in Fig. 3a,b. These results show that the activity of erlotinib was not compromised after conjugation to the nanoparticles, especially that the FeDC NPs did not show any intrinsic cytotoxicity. This retained activity could be achieved because we did not change the chemical structure of the drug during conjugation as discussed above. In addition, the selective cytotoxic effects observed with FeDC-E NPs indicate that FeDC-E NPs may target tumor cells that overexpress the EGFR rather than other cells.

### Targeting capability of the nanoparticles tested by cellular uptake experiments

To investigate the targeting capability imparted by erlotinib to the nanoparticles, we tested the enhancement of cellular uptake of nanoparticles using Prussian blue staining^26^ (Fig. 3c,d). The non-treated CL1-5-F4 cells, along with the erlotinib-treated cells did not show any blue staining spots, indicating the absence of endogenous iron in the cells and absence of precipitation of the staining reagents. The FeDC NPs-treated cells showed a few blue spots of 10.69% staining density representing the amount of FeDC NPs uptaken by the cells. The FeDC-E NPs-treated cells increased the uptake of nanoparticles 4-fold, evident from the intense blue spots observed under the microscope of 42.53% staining density. Our results regarding the low uptake of FeDC NPs are in agreement with the literature that characterizes the uptake of dextran-coated iron oxide nanoparticles^27^,^28^. However, the conjugation of erlotinib to the nanoparticles significantly increased the uptake of nanoparticles, probably because of the targeting capability of erlotinib to EGFR which is overexpressed on the cells. It is possible that the nanoparticles may aggregate on the surface of the cells without internalization, and this microscopic examination cannot exclude the extracellular localization of nanoparticles, so we carried out TEM examination of cells to confirm these uptake results.

### Targeted cellular uptake of nanoparticles detected by transmission electron microscopy

**Figure 4:**
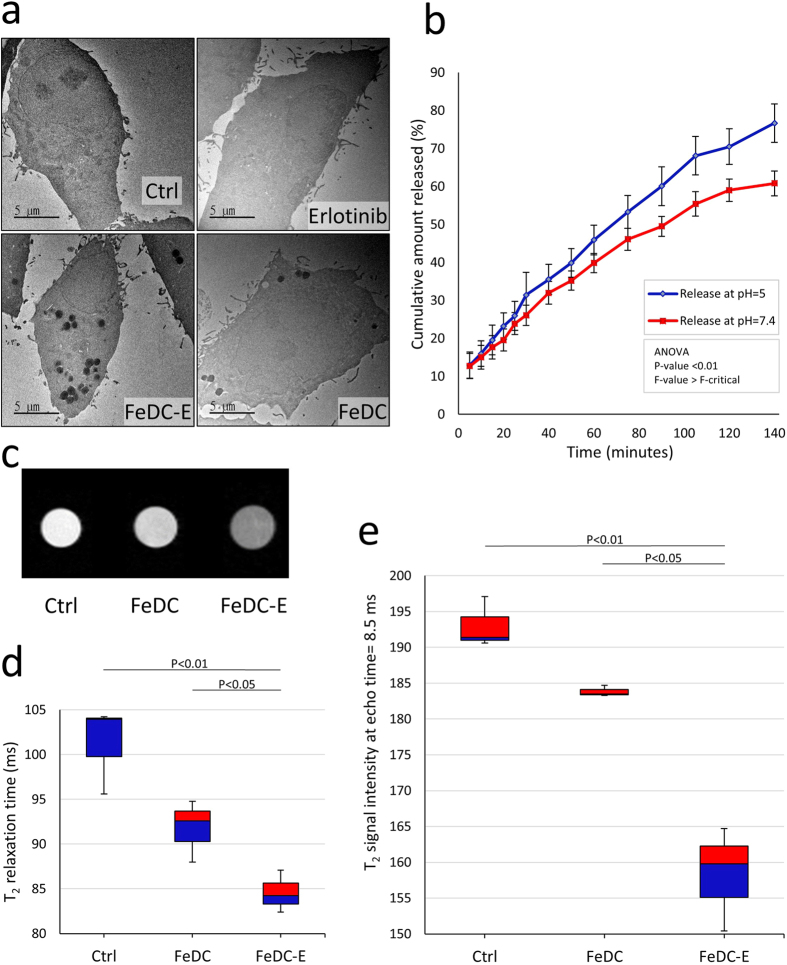
Targeted cellular uptake of nanoparticles, MRI contrast enhancement of the nanoparticles-labeled cells, and smart release system of erlotinib from nanoparticles. (**a**) Transmission electron microscopy images of CL1-5-F4 cells showing cellular uptake of nanoparticles. Nanoparticles are visible as electron-dense regions within endocytotic vesicles. The FeDC-E NPs-treated cells showed increased cellular uptake of nanoparticles compared to the FeDC NPs-treated cells due to the targeting capability of erlotinib to EGFR imparted to the FeDC-E nanoparticles. (**b**) Release profiles of erlotinib from the erlotinib-conjugated nanoparticles (FeDC-E NPs) in the extracellular environment mimicking fluid (pH = 7.4) and intracellular endocytotic mimicking fluid (pH = 5) at 37 °C. The FeDC-E NPs showed preferential release of erlotinib into the endocytotic mimicking fluid with a higher rate than into the extracellular mimicking fluid. (**c**) T_2_-weighted MRI images of CL1-5-F4 cells post-treatment with the nanoparticles. (**d**) Corresponding T_2_ relaxation times presented in a box plot. (**e**) Corresponding T_2_ signal intensities at 8.5 ms echo time where the difference in intensities between groups were maximum, presented in a box plot. FeDC-E NPs produced significant decrease of the MRI relaxation times and the MRI signal intensities of treated cells compared to the non-targeted control nanoparticles (FeDC NPs). The P-values for (**d**,**e**) were calculated using the t-Test method assuming unequal variances. Ctrl: non-treated cells, FeDC: cells treated with dextran-coated iron oxide nanoparticles and FeDC-E: cells treated with the erlotinib-conjugated dextran-coated nanoparticles.

To detect the cellular localization of nanoparticles and to confirm the cellular uptake results obtained from the Prussian blue experiments, we examined CL1-5-F4 cells of each treatment group by TEM (Fig. 4a). Images of the non-treated cells and the erlotinib-treated cells showed clear cell and nuclear membranes without any electron-dense regions. The FeDC NPs-treated cells showed few electron-dense endocytotic vesicles enclosing the nanoparticles (high electron density is due to the iron oxide core of nanoparticles). The FeDC-E NPs-treated cells exhibited similar electron-dense endocytotic vesicles of the nanoparticles but in higher quantities than the FeDC NPs-treated cells. This indicates that erlotinib increased the uptake of nanoparticles, supporting our previous findings. These TEM findings indicate that uptaken nanoparticles are localized within the endocytotic vesicles of cells with absence of extracellular aggregation of nanoparticles, supporting the Prussian blue staining results. Moreover, this confirms that conjugating erlotinib to the nanoparticles could significantly increase the cellular uptake of nanoparticles (4-fold increase as determined by Prussian blue experiments) due to the targeting capability imparted by erlotinib to the nanoparticles.

### pH dependent smart release system of erlotinib from the nanoparticles

Based on our findings that FeDC-E NPs are uptaken into the endocytotic vesicles of treated cells, and that the physiological pH of body fluids and extracellular environments is 7.4, while that of the intracellular late endosomes is about 5^29^,^30^, we designed the FeDC-E NPs to release the loaded erlotinib preferentially intracellularly into the endocytotic compartments and to reduce the release into the body fluids and extracellular environments. In order to test the efficiency of this smart release system, we tested the release of erlotinib from the FeDC-E NPs in physiological environments mimicking fluids according to Ke *et al*.^30^. FeDC-E NPs showed cumulative erlotinib release of 76.7% after 140 minutes from the start of the release in the intracellular endocytotic mimicking fluid (pH = 5), while it showed cumulative erlotinib release of 60.8% at the same time point in the extracellular environment mimicking fluid (pH = 7.4). The release of erlotinib with respect to time in these mimic fluids was statistically significantly different (Fig. 4b). This differential release data suggests that the release of erlotinib from the FeDC-E NPs will be reduced during circulation of the nanoparticles in the blood until it reaches the tumor site, where it will be endocytosed by the cells, exposed to an environment with low pH and release the load of erlotinib at a higher rate. In other words, the FeDC-E NPs exhibit a pH-dependent smart release system that enhances the drug delivery to the tumor cells and decreases the systemic effects, optimizing the overall therapeutic outcome.

### Investigation of the targeted labeling and contrast enhancement capabilities of the nanoparticles by magnetic resonance imaging

In order to verify that our nanoparticles can be used as an effective MRI contrast agent and to confirm their targeting capability, we performed *in vitro* MRI experiments using CL1-5-F4 cells (Fig. 4c) and measured the change in T_2_ relaxation times and signal intensities after different nanoparticle treatments. The non-treated cells showed median T_2_ relaxation time of 103.9 ms, while the FeDC NPs-treated cells showed median T_2_ relaxation time of 92.5 ms (Fig. 4d), this demonstrates that the amount of iron taken up by the cells could produce contrast in the MRI. Interestingly, treating the cells with the FeDC-E NPs decreased the median T_2_ relaxation time significantly to 84.2 ms (statistically significantly different from the FeDC NPs-treated cells) (Fig. 4d). The T_2_ signal intensities of the three treatment groups had the biggest differences at echo time 8.5 ms, where the non-treated cells showed median T_2_ signal intensity of 191.4, the FeDC NPs-treated cells showed median T_2_ signal intensity of 183.5, and the FeDC-E NPs-treated cells showed median T2 signal intensity of 159.8 (intensity of the FeDC-E NPs-treated cells was statistically significantly different from those of the non-treated and the FeDC NPs-treated cells (Fig. 4e). The differences in contrast between the three treatment groups are demonstrated in the MRI image in Fig. 4c. These results indicate that the cellular uptake and intracellular accumulation of FeDC-E NPs is sufficient to be traced by the MRI, and hence is suitable for use as a theranostic probe. Moreover, the T_2_ negative contrast enhancement imparted to cells treated with the targeted FeDC-E NPs relative to the non-targeted FeDC NPs, further confirm the targeting property imparted to the nanoparticles by erlotinib conjugation.

### Inhibition of the EGFR and ERK signaling pathways by the nanoparticles

Erlotinib is a highly selective small-molecule drug, specific to the tyrosine kinase domain of the EGFR that can effectively inhibit the EGFR signaling pathway^4^,^5^,^7^. EGFR is involved in many downstream signaling pathways related to carcinogenesis. The best characterized pathway is the extracellular-signal-regulated kinase (ERK) pathway which is used as a biomarker for EGFR inhibitor action. Activation of the ERK cascades by EGFR involves signaling through the Ras, Raf and mitogen-activated protein kinase/extracellular signal-regulated kinase (MEK), and is known as the EGFR–Ras–Raf–MEK–ERK signaling pathway^31^. In CL1-5-F4 cells, activation of the ERK pathway leads to activation of the transcription factor NF-κB which plays a pivotal role in regulating the expression of many genes involved in the progression of cancer such as the invasion and migration enhancing matrix metallopeptidase 9 enzyme MMP-9^32^, the angiogenesis stimulating vascular endothelial growth factor VEGF^33^,^34^, and the X-linked inhibitor of apoptosis protein (XIAP) which is considered to be the most potent direct caspase inhibitor leading to enhanced proliferation of tumor cells^35^,^36^.

**Figure 5:**
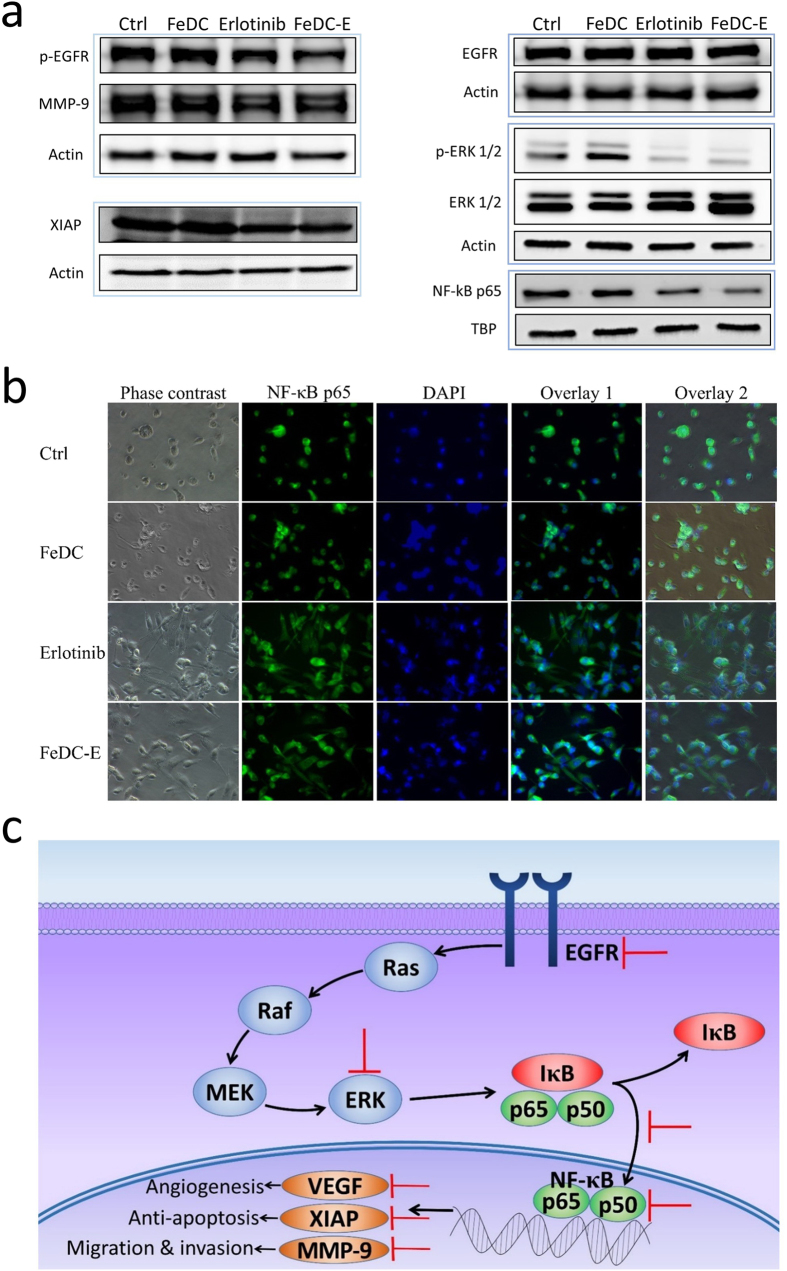
Cellular signaling pathways that may account for the biological activities observed with the treatment of CL1-5-F4 cells with the erlotinib-loaded nanoparticles (FeDC-E NPs). (**a**) Signaling pathway components of CL1-5-F4 cells altered due to treatment with nanoparticles (determined by western blot experiments). FeDC-E NPs, similar to erlotinib, inhibited the expression of p-EGFR, p-ERK 1/2, NF-κB p65 subunit, XIAP and MMP-9, and did not affect the expression of EGFR and ERK. Ctrl: non-treated cells, FeDC: cells treated with the dextran-coated iron oxide nanoparticles and FeDC-E: cells treated with the erlotinib-conjugated dextran-coated nanoparticles. (**b**) Immunocytochemical fluorescent micrographs of CL1-5-F4 cells. Treatment with either erlotinib or erlotinib-loaded nanoparticles (FeDC-E NPs) inhibited the nuclear translocation of NF-κB, resulting in the inhibition of the NF-κB pathway. Green color denotes the NF-κB p65 subunit and blue color denotes the nuclei of cells. (**c**) Proposed molecular mechanism of action of FeDC-E NPs. FeDC-E NPs inhibited the activities of EGFR, ERK and NF-κB (EGFR–ERK–NF-κB signaling pathways), resulting in the suppression of the cancer promoting proteins such as MMP-9, XIAP, and VEGF. Red T-shaped lines indicate the pathway components that were inhibited, as evident from western blot and immunocytochemical imaging experiments.

In order to investigate the alteration in cellular signaling through these pathways following treatment with FeDC-E NPs, we examined the expression of EGFR and its phosphorylated form in CL1-5-F4 cells by western blot experiments and found that the non-treated cells express both forms of the receptor (EGFR and p-EGFR) which is in agreement with the literature^20^. Erlotinib treatment did not affect the expression of EGFR, but it inhibited the phosphorylation of EGFR, which is the main mechanism of this drug as a tyrosine kinase inhibitor. Similarly, the FeDC-E NPs-treated cells showed unaltered expression of EGFR and inhibited the phosphorylation of EGFR, indicating that FeDC-E NPs are capable of inhibiting the EGFR signaling. Consequently, the downstream ERK signaling was inhibited following inhibition of EGFR. This was evident from the decrease in expression of phosphorylated ERK (p-ERK 1/2) in the erlotinib and FeDC-E NPs-treated groups compared to the non-treated and the FeDC NPs-treated groups. Expression of ERK (ERK 1/2) was not changed among the four treatment groups (Fig. 5a).

### Inhibition of the NF-κB signaling pathway and its regulated tumor promoting proteins by the nanoparticles

The transcription factor NF-κB is a heterodimeric complex of proteins of the Rel family, including the RelA (p65) subunit which is one of most widespread dimer subunits of NF-κB found in cells^37^. NF-κB is typically located in the cytoplasm in its inactive state, bound to the IκB inhibitory proteins. Following activation, the inhibitory IκB is phosphorylated and degraded, allowing NF-κB to translocate to the nucleus and bind to promoter regions of the DNA, initiating transcription and expression of the NF-κB target genes^38^,^39^,^40^. To investigate subsequent inhibition of the NF-κB pathway downstream of EGFR and ERK, we detected the amounts of NF-κB p65 subunit in nuclear extracts of CL1-5-F4 cells by western blotting and tested the localization of the NF-κB p65 subunit from the cytoplasm to the nucleus of CL1-5-F4 cells using immunocytochemical fluorescent imaging experiments. The non-treated cells showed high levels of the NF-κB p65 subunit in the nuclear fractions of tested cells as determined by western blot experiments, indicating high NF-κB activity, which is in agreement with a previous report^32^. The FeDC NPs-treated cells showed similar results to those of the non-treated cells, indicating that the nanoparticles have no effect on the NF-κB pathway without the drug. Both the erlotinib- and FeDC-E NPs-treated groups showed reduced amounts of the nuclear NF-κB p65 subunit, indicating that the NF-κB pathway is inhibited following treatment (Fig. 5a). Along the same line, the immunocytochemical imaging showed that the NF-κB p65 subunit resides in the nucleus in the non-treated as well as the FeDC NPs-treated CL1-5-F4 cells demonstrating an activate NF-κB pathway. However, the erlotinib- as well as the FeDC-E NPs-treated cells showed clear accumulation of the NF-κB p65 subunit in the cytoplasm rather than in the nuclei of examined cells (inhibited nuclear translocation of the NF-κB), indicating inhibited NF-κB activity following treatment (Fig. 5b).

Next, we examined the effect of nanoparticle treatment on expression of the proteins regulated by the NF-κB transcriptional activity and have significant roles in the progression of cancer such as XIAP and MMP-9 (Fig. 5a). Treatment of CL1-5-F4 cells with either erlotinib or FeDC-E NPs suppressed expression of XIAP compared to the non-treated and FeDC NPs-treated groups. Inhibiting XIAP has been an attractive strategy to treat cancer, because XIAP can bind directly to caspase-9, caspase-7 and caspase-3 arresting activation of the caspase cascade and preventing cancer cells death^35^. Furthermore, we were particularly interested in investigating the expression of the MMP-9 enzyme in CL1-5-F4 cells following treatment with the nanoparticles, as these highly invasive and migrative cells harbor overexpressed MMP-9 enzyme which is capable of degrading the type IV collagen of the cellular basement membrane and extracellular matrix, promoting their invasion and migration^32^. Treating CL1-5-F4 cells with either erlotinib or FeDC-E NPs suppressed expression of MMP-9 compared to the non-treated and FeDC NPs-treated groups. To detect the subsequent effect of reduced MMP-9 expression on the migrative and invasive potential of CL1-5-F4 cells, we performed migration and invasion experiments as illustrated later in this study.

### Molecular signaling pathways that may account for the potent anticancer activities of the nanoparticles

Based on the above results regarding the molecular signaling components and pathways altered due to nanoparticle treatment, we concluded that the FeDC-E NPs formulation exert its potent anticancer activities through inhibiting the tyrosine kinase domain of EGFR, resulting in the inhibition of the downstream ERK signaling pathway. Inhibition of ERK results in suppression of the NF-κB activity by preventing translocation of NF-κB from the cytoplasm to the nucleus. This will downregulate the NF-κB target genes that play a role in cancer promotion and progression. Among the proteins that were suppressed subsequent to inhibition of the NF-κB transcriptional activity, were XIAP (antiapoptosis) and MMP-9 (migration and invasion). An illustration of this signaling pathway is presented in Fig. 5c.

### Suppression of the migration and invasion capabilities of CL1-5-F4 cells following treatment with nanoparticles

**Figure 6:**
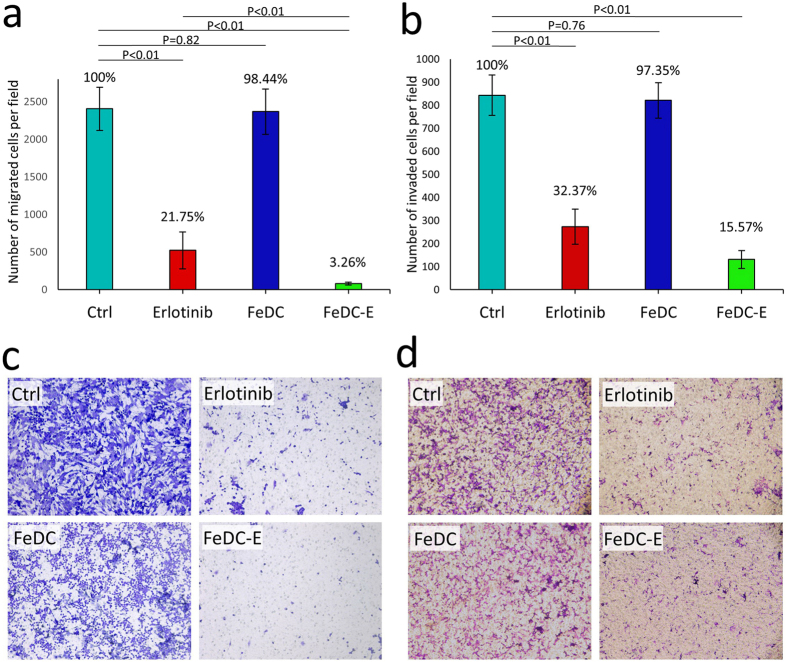
Migration and invasion of CL1-5-F4 cells. **(a)** Migration of CL1-5-F4 cells tested by the transwell assay, and presented as percentages of migrated cells. **(b)** Invasion of CL1-5-F4 cells tested by the Matrigel transwell assay, and presented as percentages of invaded cells. **(c)** Microscopical images of the cells migrated to the lower side of the membrane. **(d)** Microscopical images of the cells invaded through the Matrigel to the lower side of the membrane. Treating cancer cells with either FeDC-E NPs or erlotinib significantly inhibited the migration and invasion capabilities of cells. FeDC-E NPs showed more inhibition activities than erlotinib. (Ctrl) non-treated cells, (FeDC) cells treated with dextran-coated iron oxide nanoparticles, (FeDC-E) cells treated with the erlotinib-conjugated dextran-coated nanoparticles. Percentages of the migrated and invaded cells were quantified using ImageJ software, and the P-values were calculated using the t-Test method assuming unequal variances.

In many types of cancer, activated MMP-9 expression induces migration and invasion of the cells^41^,^42^,^43^,^44^,^45^. Our results showed that treating CL1-5-F4 cells with FeDC-E NPs decreased expression of MMP-9, so we aimed to test the subsequent effects on the migration and invasion capabilities of this highly invasive and migrative subline using the transwell migration and invasion assays (Fig. 6). CL1-5-F4 cells showed high levels of migration to the underside of the transwell membranes, in agreement with previous reports^22^,^23^,^32^. The FeDC NPs-treated cells showed less migration potential than the non-treated cells; however, the difference was slight and not statistically significant. The erlotinib-treated cells showed significant decrease in the migration potential of cells (21.75% of the non-treated cells), while treating the cells with FeDC-E NPs almost completely inhibited the migration potential of cells (3.26% of the non-treated cells) (Fig. 6a,c). These findings are in line with our results concerning the MMP-9 expression. Since the FeDC-E NPs effectively inhibited the migration of cells, we investigated their effect on the invasion capabilities of cells as well. To test the invasion of cells, we used the Matrigel-coated transwell invasion experiments. In agreement with our previous findings, the non-treated CL1-5-F4 cells showed high levels of invasion through the Matrigel to the lower side of the transwell membrane. While the FeDC NPs-treated cells showed less invasion potential, the decrease was slight and not statistically significantly different from the non-treated cells. The erlotinib-treated cells showed significant reduction in their invasion potential evident by the reduction in the percentage of invaded cells to 32.37% of the non-treated cells. Interestingly, treating the cells with FeDC-E NPs decreased their invasion potential more than erlotinib (15.57% of the non-treated cells) (Fig. 6b,d). Together, we conclude that FeDC-E NPs significantly suppressed the invasion and migration capabilities of CL1-5-F4 cells more than erlotinib at the same concentrations. Similar effects have been observed in the C6 rat glioma cells treated with either chlorotoxin alone or chlorotoxin attached to iron oxide nanoparticles, where the iron oxide-chlorotoxin nanoparticle-treated cells showed significant reduction in the migration and invasion potential of cells compared to the chlorotoxin-treated cells^46^.

### *In vivo* therapeutic efficiency of the nanoparticles

**Figure 7:**
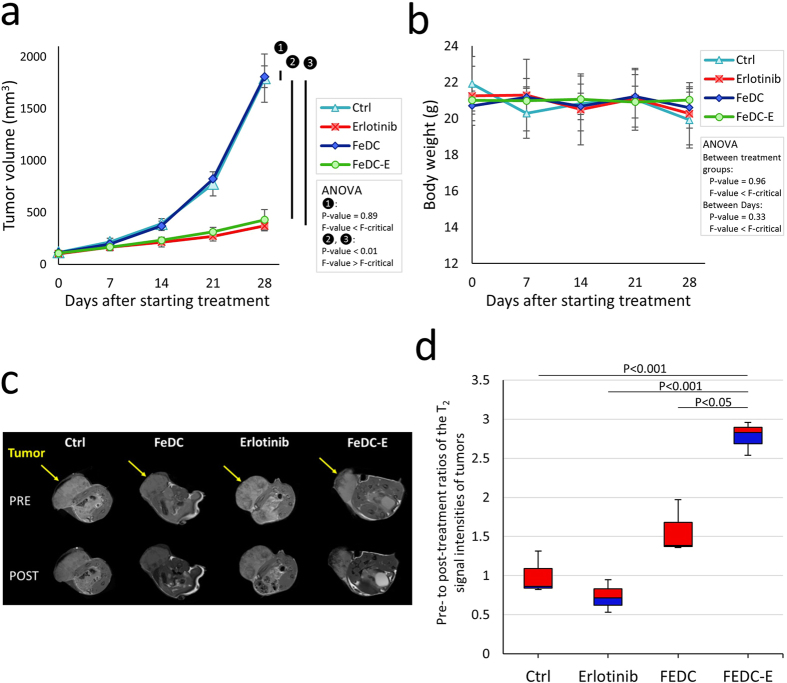
*In vivo* validation of the antitumor, MRI signal enhancement, and targeting capabilities of the nanoparticles using male BALB/c nude mice bearing xenograft implants of CL1-5-F4 cells. (**a**) Tumor volumes of the non-treated mice (Ctrl), erlotinib-treated mice, FeDC NPs-treated mice, and FeDC-E NPs-treated mice through the course of treatment, showing the significant antitumor activities of FeDC-E NPs and erlotinib compared to the control groups. The tumor dimensions were measured using a digital caliper. Please refer to text for the treatment regimen. (**b**) Body weights of the treated mice throughout the experiment period, showing no indication of toxicity to any of the treatment groups. (**c**) Representative T_2_-weighted MRI images of the tested mice acquired pre- and post-treatment for the four groups of mice (arrows point to the tumors). Significant decrease in the MRI signal intensities within tumors of the FeDC-E NPs-treated mice compared to the other treatment groups was observed following initial treatment. (**d**) Change in the T_2_-weighted MRI signal intensities within tumors following treatment, presented as ratios of the pre-treatment: post-treatment normalized MRI signal intensities. The FeDC-E NPs-treated mice showed significant drop in the MRI signal intensities within tumors compared to the control non-targeted FeDC NPs-treated group, indicating enhanced targeted accumulation of FeDC-E NPs in the tumors. The P-values for (**d**) were calculated using the t-Test method assuming unequal variances.

To confirm our *in vitro* observations regarding the cytotoxic effects of FeDC-E NPs and to investigate their *in vivo* antitumor activity, we used a tumor xenograft model of CL1-5-F4 cells in BALB/c nude mice with four treatment groups (Ctrl, Erlotinib, FeDC NPs, FeDC-E NPs). FeDC NPs-treated mice did not show any statistical significant difference in tumor volumes compared to the non-treated group, indicating that the nanoparticles have no intrinsic activity to inhibit tumor growth. On the other side, erlotinib-treated mice showed significantly inhibited tumor growth compared to the non-treated group, indicating that the tumor cells are sensitive to erlotinib. Along the same line, treating the mice with FeDC-E NPs significantly inhibited the growth of tumors compared to the non-treated group, indicating that the nanoparticles have appropriate therapeutic properties (Fig. 7a). Although the effect of FeDC-E NPs on tumor inhibition was slightly less than that of erlotinib, the difference was not statistically significant, indicating that the nanoparticles retained the therapeutic activity of erlotinib after the formulation process. No signs of toxicity were observed among the treatment groups (no statistical significant changes in the mice body weights were observed between the treatment groups throughout the experiment period) (Fig. 7b), highlighting the biocompatible nature of our nanoparticles.

### Magnetic resonance enhancement and targeting capabilities of the nanoparticles tested by *in vivo* non-invasive magnetic resonance imaging

To determine whether the MRI contrast enhancement observed with FeDC-E NPs in our *in vitro* experiments could be translated *in vivo*, and to further confirm the targeting capabilities of such nanoparticles, we used male BALB/c nude mice bearing xenograft implants of CL1-5-F4 cells for the *in vivo* non-invasive MRI experiments. Four treatment groups (Ctrl, Erlotinib, FeDC NPs, FeDC-E NPs) were used in these experiments (Fig. 7c). Normalized T_2_-weighted MRI signal intensities of the tumors (regions of interest (ROI)) were quantified pre- and post-treatments, and the changes in signal intensities for every treatment group were expressed as pre-treatment: post-treatment signal ratios and presented in Fig. 7d. Normalized signal intensities of tumors of the non-treated and erlotinib-treated groups did not show significant changes following treatments. FeDC NPs- and FeDC-E NPs-treated groups showed about 1.6-fold and 2.8-fold decrease in the mean normalized signal intensities, respectively. Decrease in the normalized signal intensity of FeDC-E NPs-treated group showed significant statistical differences from those of the other three treatment groups, indicating enhanced targeted accumulation of FeDC-E NPs in the tumors compared to the non-targeted FeDC NPs. Such *in vivo* targeted MRI contrast enhancement further supports our nanoparticles as a potential theranostic probe that could be translated into clinical applications.

## Conclusion

This is the first study to use erlotinib as a small-molecule targeting agent for nanoparticles. The theranostic nanoparticles presented in this study exhibited significant smart therapeutic and targeting properties against highly invasive and migrative cancer cells and could be monitored by MRI. Moreover, the nanoparticles suppressed the EGFR–ERK–NF-κB signaling pathways as well as the expression of the related tumor promoting proteins MMP-9 and XIAP, and subsequently inhibited the migration and invasion capabilities of cancer cells. Targeting, therapeutic, and imaging capabilities of our nanoparticles were confirmed *in vivo* using xenograft-bearing mice. Finally, the ultra-small size of our nanoparticle formulation makes it an excellent candidate for MRI of metastatic brain tumors.

## Methods

### Synthesis of the nanoparticles

MION were synthesized using the alkaline co-precipitation method^15^,^16^,^17^. Briefly, ice cold ammonia was added to an aqueous solution of FeCl_3_.6H_2_O, FeCl_2_.4H_2_O and dextran, and stirred on ice in an inert atmosphere, heated to 85 °C for 1 hour, left to cool while stirring overnight, and dialyzed extensively against Milli-Q water for 3 days. Dextran coating was crosslinked using Epichlorohydrin/NaOH followed by extensive dialysis against Milli-Q water for 3 days producing stable FeD NPs. FeD NPs were treated with ammonia to produce primary amino groups on the surface of the nanoparticles, followed by extensive dialysis against Milli-Q water for 3 days producing FeDN NPs. FeDN NPs were reacted with succinic anhydride in DMSO under buffered conditions (pH = 8.5; 0.1 M NaHCO_3_) followed by extensive dialysis against Milli-Q water for 3 days, the solution was then buffered by addition of 4-Morpholineethanesulfonic acid and NaCl producing FeDC NPs. Finally, erlotinib was added in excess to the FeDC NPs, sonicated and stirred for 2 days under a controlled temperature of 25 °C. The solution was then separated from the non-conjugated erlotinib and sterilized using 0.22 μM MCE filters producing FeDC-E NPs (Fig. 1a).

### Attenuated total reflectance fourier transform infrared spectroscopy

The ATR FT-IR spectra of nanoparticles were obtained using a Nicolet iS5 FT-IR spectrometer, Thermo Scientific, USA. Samples were analyzed using the machine’s iD3 ATR accessory in the mid-infrared region from 4000 cm^−1^ to 600 cm^−1^.

### Zeta potential and dynamic light scattering

The surface charges of the nanoparticles were determined using the ZetaPlus zeta potential analyzer, Brookhaven Instruments Corporation, USA. The hydrodynamic sizes of the nanoparticles were determined by measuring the DLS using a Horiba LB-500 particle size distribution analyzer, Japan.

### Microwave plasma - atomic emission spectrometry

The amount of iron in the nanoparticles was determined quantitatively by measuring the MP-AES of samples using Agilent 4200 MP-AES machine. Standard curve of MP-AES intensities of known standard concentrations of iron was constructed and used to calculate the unknown concentrations of iron in the nanoparticle samples.

### High-performance liquid chromatography for estimation of erlotinib concentration

HPLC was conducted according to a published study with modification^47^, using a Hitachi L-2000 series system with UV detector (L-2400, Hitachi) equipped with a C18 reverse-phase column (XBridge, Waters). The mobile phase was potassium dihydrogen orthophosphate, acetonitrile and methanol (65:25:10 V/V) at pH 2.9, run at a flow rate of 1.5 mL/min and the effluents were monitored at UV wavelength of 246 nm.

### Transmission electron microscopy of the nanoparticles

TEM images were acquired using JEOL JEM-2010 and JEOL EM-1230 microscopes (Japan) operated between 80–200 kV accelerating voltages. Samples were mounted on TED PELLA grids. For the cellular uptake of nanoparticles, 80% confluent cells were treated with the assigned treatments (12.8 μM erlotinib or equivalent of FeDC-E NPs or FeDC NPs (equal to the volume and iron concentration of the used FeDC-E NPs)) and incubated for 24 hours. Next, cells were washed with PBS, detached from the plates by Trypsin/EDTA, fixed with 2.5% glutaraldehyde overnight, washed, stained with 1% osmium tetroxide for 2 hours, dehydrated, embedded in Epon resin, sectioned with a diamond knife and mounted on the grids.

### Cell viability assay

Viability of cells was tested by the WST-1 assay^48^. Briefly, either 3 × 10^3^ CL1-5/F4 cells or 2 × 10^3^ Jurkat cells were seeded into 96-well plates and incubated for 24 hours. Erlotinib or FeDC-E NPs were added to the cells to give final erlotinib concentrations of 3, 6, 10, 12, 14, 16, 24, 48 μM. FeDC NPs were added in equal volumes to those of the FeDC-E NPs. Then the cells were incubated for a further 24 hours after which WST-1 cell proliferation reagent (Roche Diagnostics, Germany) was added in 10 μL aliquots to every well and incubated for 4 hours. The absorbances of each well were measured at 450 nm and reference wavelength 630 nm.

### Detection of iron labeled cells

Cellular uptake of nanoparticles was tested using the Prussian blue staining procedures for iron^26^. Briefly, 1 × 10^6^ cells were seeded in a 24-well plate and incubated for 24 hours then treated with the assigned treatments (12.8 μM erlotinib or equivalent of FeDC-E NPs or FeDC NPs (equal to the volume and iron concentration of the used FeDC-E NPs)) and incubated for a further 24 hours. Next, cells were washed with PBS, fixed with 10% neutral buffered formalin and stained using the Prussian Blue staining kit for iron oxide magnetic nanoparticles on cells/tissues (Ocean NanoTech, USA). The Prussian blue labeling density was quantified by ImageJ software using the formula [(blue stained area in pixels/total cells area in pixels) × 100] and presented as a percentage^49^.

### Release of erlotinib from the nanoparticles

Release of erlotinib from the FeDC-E NPs in different fluids was tested using the dialysis method^30^. Briefly, 1 mL of the FeDC-E NPs solution was placed in a dialysis bag of molecular-weight cutoff 12000–14000 and dialyzed against 200 mL of PBS at either pH = 7.4 or pH = 5 at 37 °C. Aliquots of 1 mL were removed at the specified time points from the PBS and replaced with fresh PBS. The amount of erlotinib was determined by HPLC. The cumulative amount of erlotinib released was calculated as^50^:


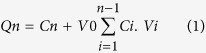
where *Qn* is the cumulative amount of erlotinib released (μg/mL) at time t (minutes), *Cn* is the drug concentration of the dissolution medium at each sampling time, *Ci* is the drug concentration of the *i* th sample, *V0* is the volume of the dissolution medium and *Vi* is the volume of the sample.

### Magnetic resonance imaging of the nanoparticle-treated cells

*In vitro* MRI experiments were performed on CL1-5-F4 cells. Briefly, 1 × 10^6^ cells were treated with either 12.8 μM erlotinib equivalent of FeDC-E NPs or FeDC NPs (equal to the volume and iron concentration of FeDC-E NPs) for 24 hours; non-treated CL1-5-F4 cells were also tested as a control group. After appropriate washing and harvesting, cells were embedded in 1% agarose in PCR tubes with all the air bubbles removed and imaged with a 7T Bruker PharmaScan MRI scanner (using a volume coil I.D. = 72 mm) (Bruker BioSpin, MA, USA). The T_2_-weighted images were acquired by spin-echo sequences with echo time (TE) = 8.5 ms, repetition time (TR) = 3000 ms, 20 echoes, flip angle (FA) = 90°, field of view (FOV) = 50 × 50 mm, resolution = 256 × 256 and slice thickness = 0.5 mm.

### Western blot experiments for protein expression

Treatment groups of 1 × 10^6^ of CL1-5-F4 cells treated with 12.8 μM erlotinib or equivalent FeDC-E NPs or FeDC NPs (equal to the volume and iron concentration of FeDC-E NPs) for 24 hours, were harvested and washed with PBS on ice. Non-treated CL1-5-F4 cells were also tested as a control group. Nuclear extracts were prepared using a Nuclear/Cytosol fractionation kit (BioVision, USA) according to the manufacturer’s protocol (briefly, appropriate buffers of the kit were used to extract the cytosolic or nuclear fractions and separated by centrifugation). Proteins were separated by electrophoresis in SDS-PAGE gels, transferred to PVDF Membranes and blocked by fat free milk. Membranes were probed with any of anti-EGFR antibody (ab52894), anti-EGFR (phospho Y1092) antibody (ab40815), anti-Erk1/2 (p44/p42) antibody clone MK12, anti-phospho-Erk1/2 (Thr202/Tyr204, Thr185/Tyr187) antibody recombinant clone AW39R, anti-NF-κB p65 antibody (ab32536), anti-MMP‑9 antibody (AF911), anti-XIAP antibody (ab21278), anti-beta Actin antibody (ab119716) or anti-TATA binding protein (TBP) antibody (1TBP18), washed, incubated with secondary antibodies coupled to horseradish peroxidase. The immunoreactive bands were visualized using Immobilon Western Chemiluminescent HRP Substrate kit. After acquiring the images of protein bands, membranes were stripped and reprobed to detect next proteins of interest (if the same membrane was to be used). Equal loading of total proteins into the wells was confirmed by probing with either beta actin or TBP antibodies.

### Immunocytochemical fluorescent imaging of cells

Inhibition of NF-κB was detected using the immunocytochemical fluorescent imaging according to previous reports^48^,^51^. Briefly, treated or untreated cells seeded on coverslips were fixed, permeabilized with Triton X-100, treated with anti-NF-κB p65 rabbit monoclonal primary antibody Abcam #ab32536, followed by Alexa Fluor 488 Donkey anti-rabbit IgG secondary antibody Biolegend #406416. Nuclei of the cells were stained with 4’,6-diamidino-2-phenylindole (DAPI). Images were acquired using a fluorescence microscope.

### Migration and invasion assays

We used the transwell chambers to test the migration and invasion of cells^22^. Briefly, 1 × 10^6^ cells/well were seeded in transwell inserts with polycarbonate membrane of 8 μm pore size and treated with either 12.8 μM erlotinib, FeDC-E NPs of equivalent amount of erlotinib or FeDC NPs (equal to the volume and iron concentration of FeDC-E NPs used), non-treated CL1-5-F4 cells were also tested as a control group. The transwell inserts were coated with 1:1 Matrigel:DMEM/F12 for the invasion experiments, or used uncoated for the migration experiments. After incubation, cells on the upper chamber of the well were removed and membranes were fixed, stained with hematoxylin, air-dried, examined with a microscope and quantified by ImageJ software.

### Xenograft model

Animal experiments presented in this study were carried out in accordance with the guidelines and regulations of the Institutional Animal Care and Use Committee (IACUC) at Taipei Medical University. All animal experiments presented in this study were reviewed and approved by the IACUC at Taipei Medical University (approval number: LAC-2014-0107), and complied with the “3Rs” principle of animal research. Eight-week-old male BALB/c nude mice were purchased from the National Laboratory Animal Center, Taiwan. Suspensions of 2.5 × 10^6^ of CL1-5-F4 cells were implanted subcutaneously into the left hind leg of the mice. When the tumor volume reached 100-120 mm^3^, the mice were divided randomly into four treatment groups (Ctrl: no treatment control injected with PBS, Erlotinib: treated with an erlotinib dose of 10 mg/Kg of body weight^52^, FeDC-E: treated with FeDC-E NPs having erlotinib content of 10 mg/Kg of body weight, FeDC: treated with FeDC NPs of equivalent Fe concentration as the used FeDC-E dose). The assigned treatments were given five times weekly by appropriate intravenous administration. Mice body weights were monitored using digital scale, and tumor dimensions were measured using digital caliper. Tumor volumes were calculated using the formula [Tumor volume = 0.523 × Length × Width^2^]^53^.

### *In vivo* magnetic resonance imaging

Untreated male BALB/c nude mice bearing xenograft implants of CL1-5-F4 cells were used for the *in vivo* MRI imaging of the mice. T_2_-weighted scans of the mice were performed using 7T Bruker PharmaScan MRI scanner under isoflurane anesthesia before receiving any treatment. Next, the mice received the assigned treatments as mentioned earlier (Ctrl, Erlotinib, FeDC NPs, FeDC-E NPs), euthanized 15 minutes after receiving the treatments, and scanned under the same conditions of the MRI scanner used before receiving treatments. T_2_-weighted images were acquired by spin-echo sequences with TR = 3000 ms, TE = 8.5 ms, FOV = 3.5 × 3.0 cm, matrix size = 128 × 128, 6 slices and slice thickness of 1 mm. Signal intensities of ROI were quantified using Bruker ParaVision 6.0.1 software system for a fixed area of 0.06 cm^2^ for all tumors, and normalized to intensities of self soft tissue (muscle) which showed little change pre- and post-treatment.

### Statistical analysis

Data are presented as means ± SD. Statistical analysis of the results was calculated using the two-way analysis of variance (ANOVA) with replication method, and the t-Test assuming unequal variances method to calculate the two-tail P-values of samples. The used statistical analysis methods are mentioned on every figure and/or figure legend.

### Materials

CL1-5-F4 and Jurkat cell lines were provided by professor Chia-Ling Hsieh, Taipei Medical University. CL1-5-F4 cell line was cultured in DMEM/F12 (1:1 mixture) medium, supplemented with 10% FBS and 1% penicillin/streptomycin. Jurkat cell line was cultured in RPMI 1640 Medium, supplemented with 10% FBS and 1% penicillin/streptomycin. Dextran from *Leuconostoc mesenteroides* (average MW 9000–11000), iron(III) chloride hexahydrate, iron(II) chloride tetrahydrate, ammonia solution, epichlorohydrin, succinic anhydride and Corning Spin-X UF concentrators 5K MWCO were bought from Sigma-Aldrich. Erlotinib was bought from Cayman Chemical, USA. Spectra/Por Dialysis Membrane 12–14 kD MWCO and closures were bought from Spectrumlabs, USA. Sterilized syringe driven filters, 30 mm, 0.22 μm, MCE, were bought from Advangene, USA.

## Additional Information

**How to cite this article**: Ali, A. A. A. *et al*. Erlotinib-Conjugated Iron Oxide Nanoparticles as a Smart Cancer-Targeted Theranostic Probe for MRI. *Sci. Rep.* **6**, 36650; doi: 10.1038/srep36650 (2016).

**Publisher’s note:** Springer Nature remains neutral with regard to jurisdictional claims in published maps and institutional affiliations.

## Supplementary Material

Supplementary Video

Supplementary Information
