# Supplementary material for: Erlotinib-Conjugated Iron Oxide Nanoparticles as a Smart Cancer-Targeted Theranostic Probe for MRI
Source: Sci Rep. 2016 Nov 11;6:36650. doi: 10.1038/srep36650 (PMC5105135; doi:10.1038/srep36650)
Supplement: Supplementary Information [file srep36650-s2.pdf]

Supplementary Information of

Erlotinib-Conjugated Iron Oxide Nanoparticles as a Smart Cancer-Targeted Theranostic Probe for MRI

Ahmed Atef Ahmed Ali[1,2], *Fei-Ting Hsu*[3,4], *Chia-Ling Hsieh*[5], *Chia-Yang Shiau*[6], *Chiao-Hsi Chiang*[2], *Zung-Hang Wei*[7], *Cheng-Yu Chen*[3,4*], *Hsu-Shan Huang*[1,2,8*]

[1] Molecular and Cell Biology, Taiwan International Graduate Program, Institute of Molecular Biology, Academia Sinica, Taipei 115, Taiwan.

[2] Graduate Institute of Life Sciences, National Defense Medical Center, Taipei 114, Taiwan.

[3] Department of Medical Imaging, Taipei Medical University Hospital, Taipei 110, Taiwan.

[4] Translational Imaging Research Center, College of Medicine, Taipei Medical University, Taipei 110, Taiwan.

[5] The Ph.D. Program for Translational Medicine, College of Medical Science and Technology, Taipei Medical University, Taipei 110, Taiwan.

[6] Graduate Institute of Medical Sciences, National Defense Medical Center, Taipei 114, Taiwan.

[7] Department of Power Mechanical Engineering, National Tsing Hua University, Hsinchu 300, Taiwan.

[8] Graduate Institute for Cancer Biology & Drug Discovery, College of Medical Science and Technology, Taipei Medical University, Taipei 110, Taiwan.

* Corresponding authors:

Cheng-Yu Chen, sandychen@tmu.edu.tw or Hsu-Shan Huang, huanghs99@tmu.edu.tw

Supplementary Video S1. Magnetic properties of the FeDC-E NPs solution. Application of an external magnetic field with a magnet moved the aqueous nanoparticles solution (suspended in organic immiscible solution). Please note that the iron oxide nanoparticles do not aggregate towards the magnet, but the whole solution moves instead, due to the nanoparticles stability properties. Below is a screenshot from the video, please download the video for details.

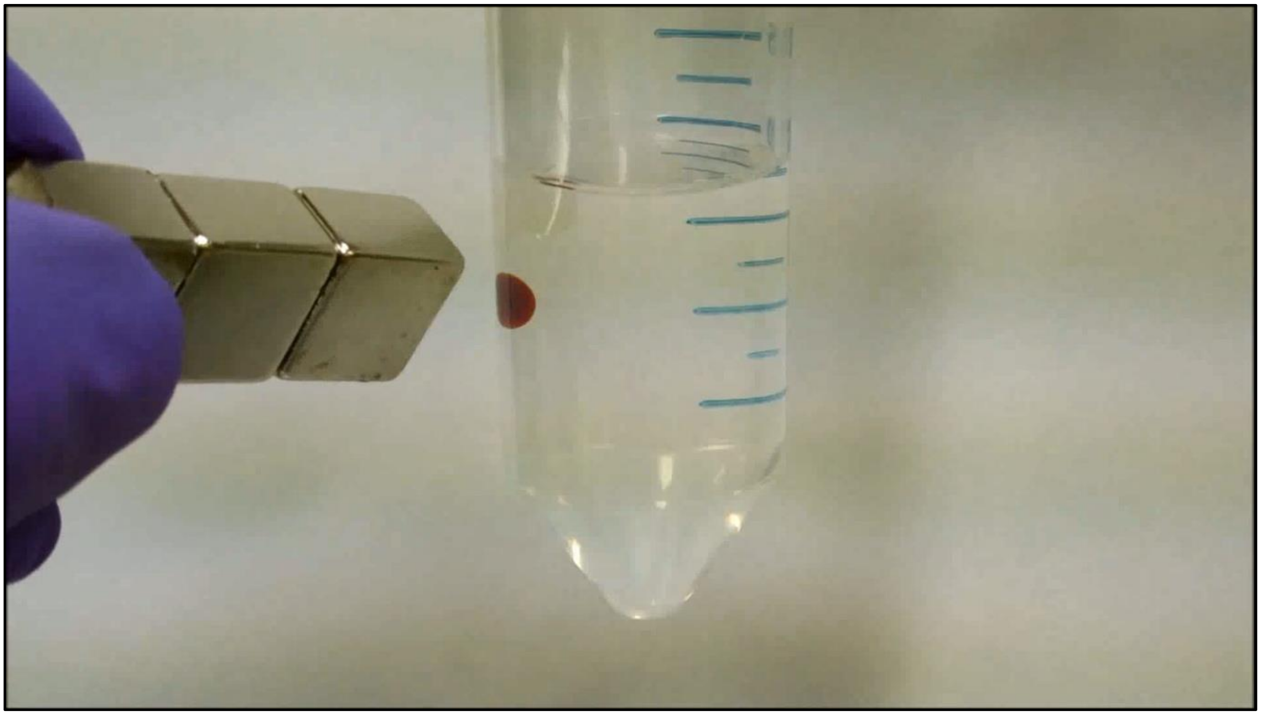

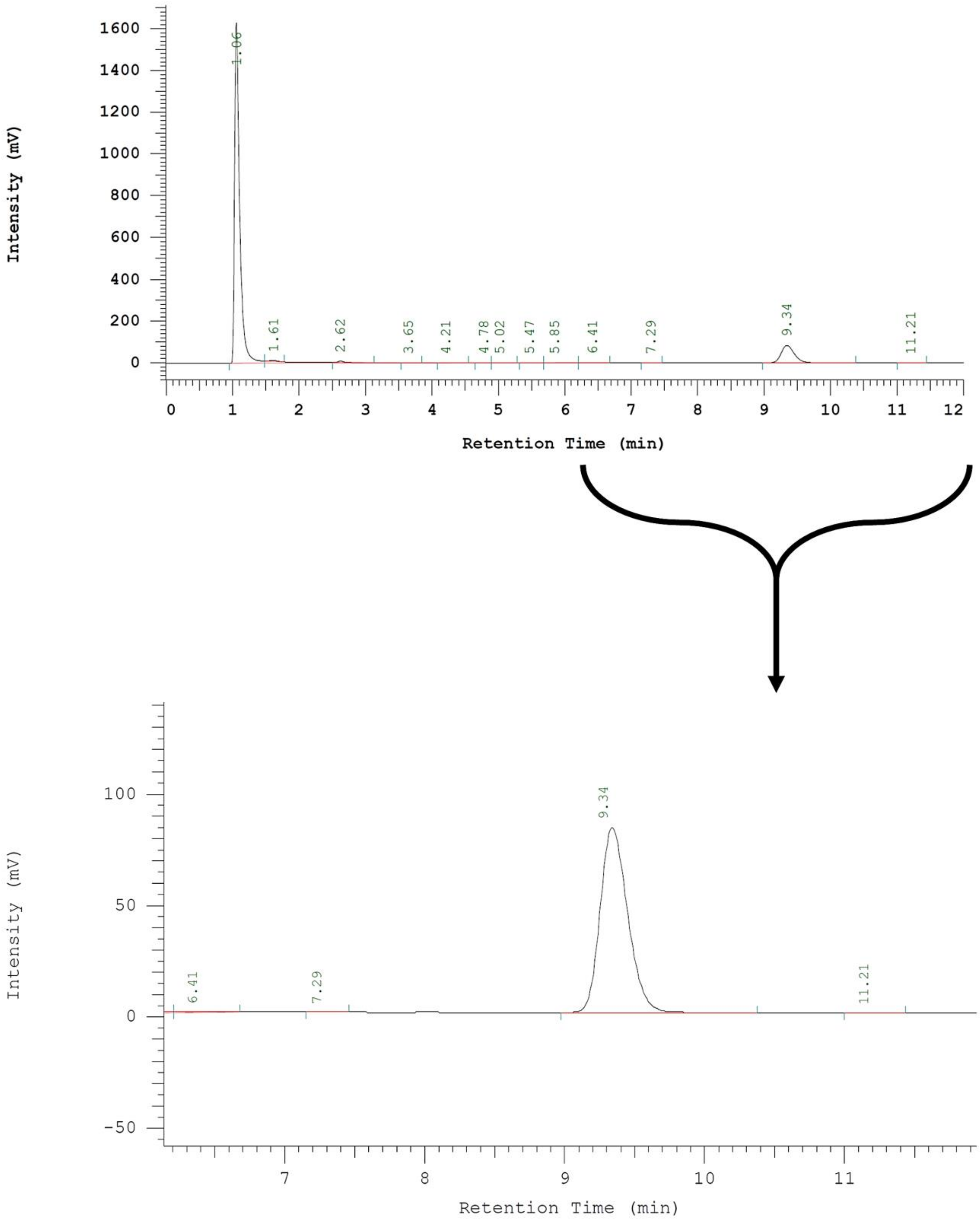

Supplementary Figure S2. Full HPLC spectrum of FeDC-E NPs diluted to ¼ of the original concentration (upper spectrum). Erlotinib peak is visible at a retention time of about 9.34 minutes. Lower spectrum shows cropped region of the erlotinib peak.

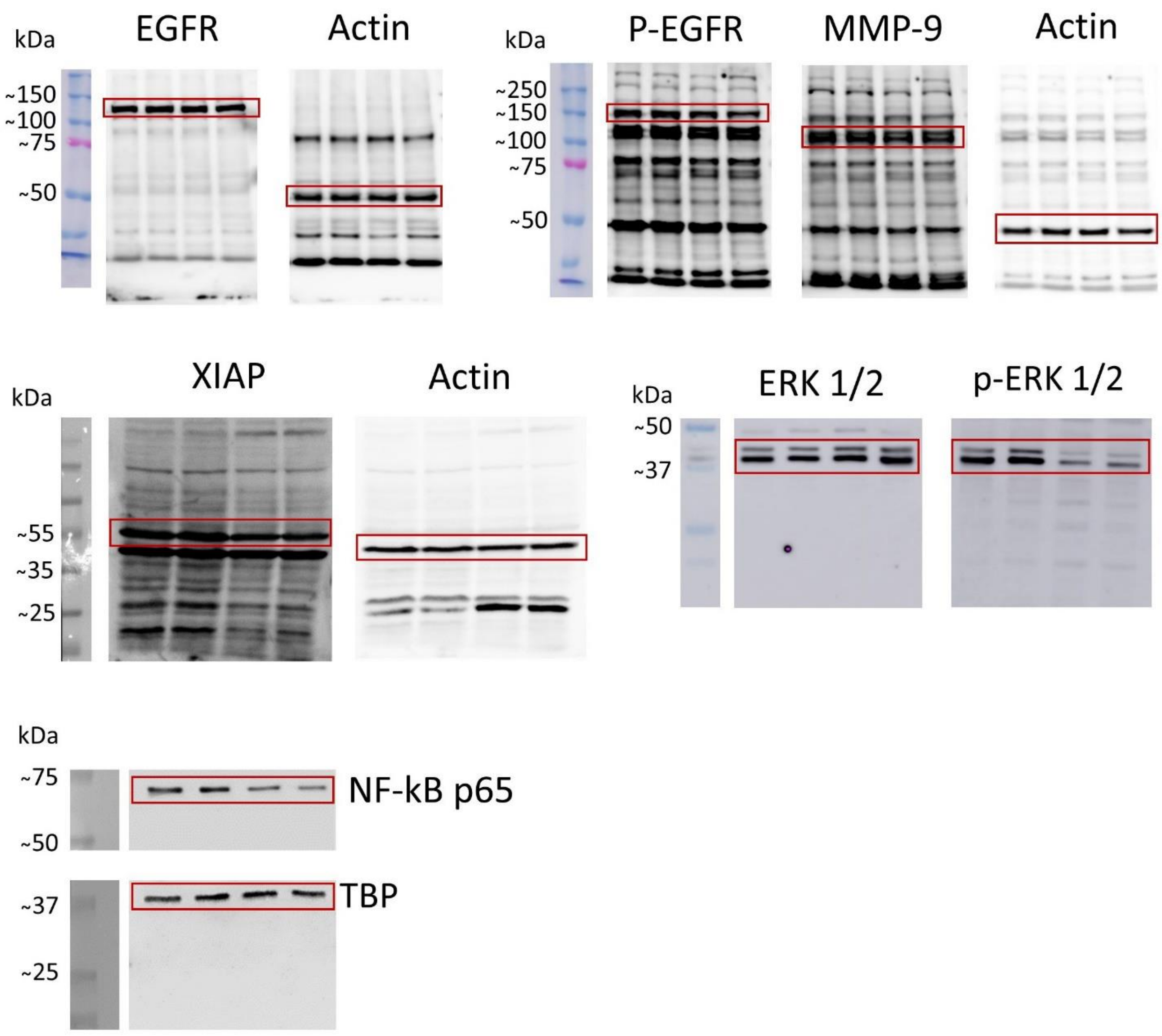

Supplementary Figure S3. Images of Western blots performed in this study.
